# From amputation to limb salvage reconstruction: evolution and role of the endoprosthesis in musculoskeletal oncology

**DOI:** 10.1007/s10195-013-0265-8

**Published:** 2013-09-22

**Authors:** John S. Hwang, Anokhi D. Mehta, Richard S. Yoon, Kathleen S. Beebe

**Affiliations:** 1 Division of Orthopaedic Oncology, Department of Orthopaedic Surgery, New Jersey Medical School, Rutgers, The State University of New Jersey, Newark, NJ 07101 USA; 2 Department of Orthopaedic Surgery, NYU Hospital for Joint Diseases, New York, NY 10003 USA

**Keywords:** Endoprosthesis, Limb salvage, Orthopaedic oncology, Bone tumors, Medical history

## Abstract

In 1943, Austin Moore developed the first endoprosthesis fashioned from Vitallium, providing the first alternative to traditional amputation as primary treatment of bone tumors. The success of the Vitallium endoprosthesis has since then led to the development of new materials and designs further advancing limb salvage and reconstructive surgery. Combined with the advent of chemotherapy use and imaging advances, conservative treatment of musculoskeletal tumors has expanded greatly. As the implantable options increased with the development of the Lewis expandable adjustable prosthesis and the noninvasive Phenix Growing prosthesis, receiving the diagnosis of a bone tumor no longer equates to automatic limb loss. Our review details the history and development of endoprostheses throughout orthopedic oncology in the treatment of musculoskeletal tumors.

## Early history

During the early 1900s, multiple unsuccessful attempts were made to incorporate the use of metal equipment into the body. These failures were primarily attributed to the researchers’ inabilities to find a suitable metal that could withstand corrosion from bodily fluid without causing an unfavorable reaction in soft tissue [1]. In 1932, Austenal Laboratories created a cobalt–chromium alloy called Vitallium specifically for use in dental implants, and, unlike previous dental alloys, Vitallium could withstand the corrosive effects of saliva. With the successful incorporation of Vitallium into dental implants, Venable et al. [1, 2] began to pursue studies investigating the effects of Vitallium on the body and discovered that this metal was inert to bodily fluids and soft tissue. Following these studies, the cobalt–chromium alloy began to be used in the orthopedic field as the preferred metal for creating plates and screws for internal fixation methods [3, 4].

**Fig. 1:**
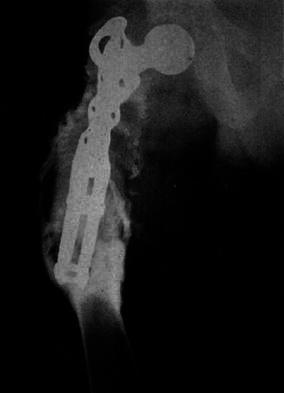
Plain radiograph of the original vitallium endoprosthesis from Moore and Bohlman [5]. Reprinted with permission from JBJS

**Fig. 2:**
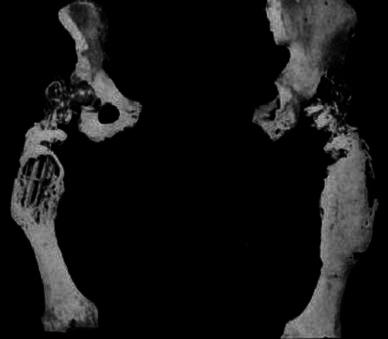
Photograph of the original vitallium endoprosthesis upon autopsy from Moore and Bohlman [5]. Reprinted with permission from JBJS

In 1943, Vitallium was used for the first metallic endoprosthesis in orthopedic oncology and, possibly, in the entire field of orthopedics. Using the alloy, Austin Moore [5] created an endoprosthesis of the proximal femur, which he implanted in a patient following resection of the proximal femur diseased by a giant cell tumor. At 1-year follow-up, remarkable results were seen. On plain radiograph and autopsic specimen, new bone formation was seen developing around the prosthesis (Figs. 1 and 2). Clinically, the patient demonstrated ambulation while carrying another man who weighed 215 pounds (97.5 kg). The next few decades were marked by the development of endoprosthetic implants created from Vitallium [6–9] and other materials—stainless steel [10], polythene [11], acrylic [12]—for treating femoral and other long-bone defects. Although there was a growing interest in the use of endoprosthetic implants for limb salvaging, endoprostheses were primarily reserved as a palliative treatment for individuals who refused amputation.

It was not until the 1970s that endoprosthetic implants began to emerge as a valuable treatment option in orthopedic oncology. This was largely due to the various advances in medical technology, specifically in the fields of radiology, pathology, chemotherapy, and radiotherapy, that allowed staging and assessing tumoral local extension and led to an increase in survival in musculoskeletal tumor patients.

## Advances in medical imaging, and materials technology

With the development of antineoplastic drugs, orthopedic physicians began to use chemotherapy for primary musculoskeletal tumors starting in the early 1970s. The use of chemotherapy in orthopedic oncology began as adjuvant therapy following removal of the primary tumor. This followed the hypothesis that chemotherapy would destroy any microscopic residual disease missed during surgery. The results were promising. Studies showed increased survival rates in patients who received adjuvant chemotherapy following bone resection [13, 14]. Physicians began to use chemotherapy in conjunction with custom-made endoprostheses in hopes of increasing survival while also salvaging the limb [15]. As patients waited as long as 8 weeks for their custom implants, physicians began to notice the adverse effects of delaying chemotherapy until after surgical treatment. This led to studies on the effects of initiating preoperative chemotherapy while a patient awaited the custom implant. These studies demonstrated that preoperative chemotherapy caused regression of primary bone tumors and increased patient survival equivalent to studies in which patients received postoperative chemotherapy [16, 17]. Currently, the most common treatment of primary bone tumors is neoadjuvant and adjuvant chemotherapy.

Imaging studies have always played a vital role in diagnosing musculoskeletal tumors. Although plain radiograph is an essential component in early diagnosis of bone tumors, it was not until the early 1970s, with the developments of computed tomography (CT) [18, 19], and magnetic resonance imaging (MRI) in the mid 1980s [20, 21], that imaging studies could better visualize bony and soft tissue tumors [22–25]. Also in the early 1970s, the emergence of skeletal scintigraphy [26] began being used in musculoskeletal oncology due to its value for assessing bone metastasis. Another important advancement was positron emission tomography (PET), which became increasingly used in oncologic patients in the 1990s—though the technology was first reported in 1973—predominantly due to its ability to visualize metastatic disease and evaluate the effects of treatment. These advanced imaging studies provided physicians the ability to make a more thorough medical and surgical management plan [27].

Two important advancements in materials is the use of titanium alloys, specifically Ti-6Al-4, and silver coating. Pure titanium has a high affinity for oxygen, but Ti-6Al-4V undergoes a process called self-passivation to create a protective film that can resist corrosion. This quality has created many uses for this alloy in orthopedic implants [28]. Additionally, silver coating is applied to prostheses to decrease infection risk: silver ions have antibacterial activity that can help reduce periprosthetic infections [29].

## From custom to modular implants

The use of endoprostheses in limb-salvaging procedures started to gain popularity in the 1970s as chemotherapy improved and total joint replacements were increasingly used. To further improve the development of custom endoprostheses in musculoskeletal oncologic limb-salvaging procedures, researchers began thorough analyses of failure modes for these prostheses, thus encouraging continuous redesign and improvement of endoprostheses.

One of the common modes of failure in early custom endoprostheses was failure of the stem component. Suboptimal fabrication during the manufacturing process and the use of alloys that could not withstand high stress were two of the early causes of stem failure [30, 31]. Improper forging, casting, or tempering left these prostheses with suboptimal fatigue strength and hardness that, in some cases, led to stem fractures. The development of stronger alloys and a more uniform manufacturing process have helped reduce the number of stem fractures. Another cause of stem failure, specifically of proximal femoral components, is design flaw as a result of limited manufacturers’ knowledge regarding stress factors these implants must withstand in order to remain intact. Early custom proximal femoral implants typically had stems that were shorter and thinner than recent implants. These early implants were unable to endure a high degree of stress. With biomechanical testing, it was discovered that larger diameters and longer stems would help decrease stress and increase mechanical strength [32–34]. Another significant cause of stem failure was detected with the use of bone cement. John Charley popularized the use of bone cement in endoprosthetic stem fixation following his research studies in the 1970s [35]. However, with the increased use of bone cement in this capacity, it became apparent that there was some loosening between the stem component and the cement. Aseptic loosening of cementless stems occurs largely due to bone resorption around the endoprostheses. In hopes of preventing osteolysis around the endoprostheses, researchers began using porous surfaces around the stems, anticipating that this interface would provide a better surface for bone ingrowth and further stabilize the endoprosthesis [36]. Furthermore, extramedullary porous coating at the stem base would provide an area for soft tissue growth, which enabled formation of a seal around the stem, thereby preventing debris-laden fluid from entering the interface [37]. This allowed for further stem stabilization and decreased the rate of aseptic loosening.

Another challenge in implant use is attaching the tendon directly to a metallic implant. A tendon directly attached to a metallic implant, with no scaffold, is held together by fibrous ingrowth, which is a weak interface. This type of attachment has <20 % of the strength of a normal tendon insertion [38]. Normally, the natural transitional zones of the tendon–bone insertion site serve as a scaffold between the tendon and bone [39]. Furthermore, inadequate attachment of tendons to implant results in decreased joint range of motion and function. This can potentially lead to prosthesis loosening and joint instability [39]. To recreate the natural scaffold that exists between tendon and bone, an enhanced tendon anchor (ETA) can be created using autogenic cancellous bone and bone marrow. This was done in an experimental canine model to create an augmentation for tendinous insertion on metallic implants. ETA can create “the normal direct tendon insertion site” [39]. Experimental animal studies indicate that the strength of reattachment is dependent upon remodeling of the bone plate [39]. In an experimental canine study by Inoue et al., a supraspinatus tendon was attached to a porous titanium prosthesis using an autogenic cancellous bone plate and marrow. At 16 weeks postoperatively, there was a 90.3 % recovery of preoperative weight-bearing capability [38]. Drawbacks to using bone autograft are a limited supply, necessity of a second surgery, and donor-site morbidity. Allograft can be used but lacks the bone-inductive properties of autograft. Recombinant human osteogenic protein-1 (rhOP-1) can be used in combination with allograft to induce cartilage and bone formation in an ETA [39]. In an experimental animal study by Higuera et al. [39], allograft in combination with rhOP-1 yielded similar results as autogenic cancellous bone and marrow. The study also showed that tendon reattachment does not have to be completed with the full original strength of the attachment in order to regain tendon functionality [39].

**Fig. 3:**
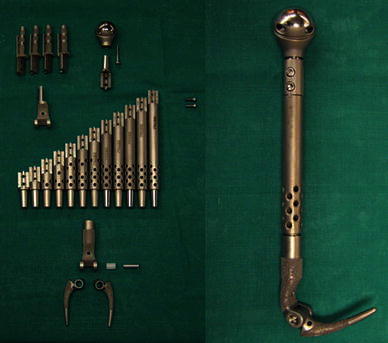
Photograph of the different modular components comprising a humeral implant to ensure the best fit for the patient from Funovics et al. [54]. Reprinted with permission from JBJS

Due to advances in the field of chemotherapy, musculoskeletal oncologists began to see an increase in the number of available candidates for limb-salvaging procedures. With this increase came the birth of current modular endoprosthetic systems that seek to create a single system, thereby simplifying bone and joint replacement procedures [36]. Prior to the development of modular systems, physicians were unable to provide an optimal-fitting prosthesis during surgery. Modular systems provided patient-specific endoprostheses that could be modified during surgery without the cost and delay of a custom prosthesis. These modular systems allowed surgeons to use components of the best size and length for the individual (Fig. 3). The components were then joined together in the operating room to create a unique and well-fitting endoprosthesis. Early systems provided treatment for the proximal femur, distal femur, proximal tibia, and proximal humerus [40–43].

Two of the most crucial characteristics essential in modular systems are reliability and simplicity. To provide a straightforward and dependable system, developers of modular endoprostheses began to use the Morse taper between joining components [44]. This taper system was developed by Stephen Morse decades before its use in endoprosthetic reconstruction. The Morse taper in orthopedic endoprostheses consists of a male end from one endoprosthetic component that can be coupled together with a female end from another component. To achieve an appropriate fit, surgeons must be cautious and diligent to ensure that the ends are clean and devoid of debris [44, 45].

Although the original groundwork on modular systems remains, biomechanical engineers continue to develop new methods to advance the system with more reliable components and fewer complications.

## Expanding the role into the pediatric population

As limb-salvage procedures became a popular method for treating malignant bone disease in adults, physicians found it difficult to treat bone tumors using standard endoprostheses in growing children. With many bone tumors occurring around the growth plates of immature patients, the use of standard endoprostheses caused limb discrepancies when these patients reached adulthood. To address this issue, researchers began to explore the idea of expandable prostheses.

The first expandable prosthesis, the Lewis expandable, adjustable prosthesis, consisted of a screw extension mechanism that was turned by making a small incision and using a chuck key to turn the screw [46, 47]. This design showed favorable results, but the need for recurrent surgeries to expand the unit posed a variety of complications for the patient, including the possibilities of nerve damage and infection. To find solutions to these problems, biomechanical engineers began exploring possibilities for internally expandable prostheses [48].

**Fig. 4:**
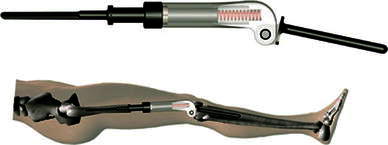
Image of REPIPHYSIS expandable prosthesis. Reprinted with permission by Wright Medical Technology, Arlington, Tennessee

**Fig. 5:**
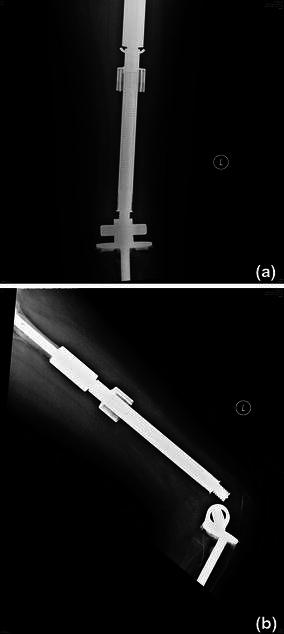
Anteroposterior (**a**) and lateral (**b**) radiographs of a pediatric patient with a REPIPHYSIS endoprosthesis

One of the earliest internally expandable prostheses was the Phenix growing prosthesis (Phenix Medical, Paris, France) [49]. This system used a preloaded spring between two titanium tubes, which was then covered by a larger polymeric tube (Fig. 4). When expansion is needed, a magnetic field is created around the spring to cause decompression. The latest model of the Phenix growing prosthesis is the REPIPHYSIS expandable limb salvage system (Wright Medical Technology, Arlington, TN, USA) (Fig. 5a, b). Although these implants spare the pediatric patient from recurrent invasive procedures, failed REPIPHYSIS prostheses have been reported in the literature; it has been suggested that unexpanded implants are at a greater risk of failure than expanded implants [50]. Using similar concepts of electromagnetic rotation in the internal mechanism, newer models of noninvasive, expandable prostheses continue to be developed. The Stanmore prosthesis (Stanmore, London, UK) consists of a motor that applies a lengthening force on the limb when placed inside of an electromagnetic coil. The coil is portable, and the procedure can be done in an outpatient setting; anesthesia or sedation is not required [51]. The MUTARS BioXpand device (Implantcast, Buxtehude, Germany) is another expandable prosthesis that uses distraction osteogenesis to lengthen the limb. It does not increase the implant length but increases the host-bone segment [52]. The goal of these models is to provide a noninvasive, expandable prosthesis that will endure until the patient reaches adulthood.

## Direction and conclusion

The evolution of machining, adjuvant therapies, and imaging techniques has contributed greatly to the advances in limb-salvage surgery. As we move further into the twenty-first century, the possibilities for advancements in limb-salvage reconstruction seem bright. Improved techniques of stem fixation and soft tissue attachment will further increase implant survivorship, improve quality of life, and continue to improve the prognosis and hope for those diagnosed with a musculoskeletal tumor [53].
